# Optical interrogation of neuronal circuitry in zebrafish using genetically encoded voltage indicators

**DOI:** 10.1038/s41598-018-23906-1

**Published:** 2018-04-16

**Authors:** Hiroaki Miyazawa, Kanoko Okumura, Kanae Hiyoshi, Kazuhiro Maruyama, Hisaya Kakinuma, Ryunosuke Amo, Hitoshi Okamoto, Kyo Yamasu, Sachiko Tsuda

**Affiliations:** 1 Division of Life Science, Graduate School of Science and Engineering, Saitama University, 255 Shimo-Okubo, Sakura-ku, Saitama City, Saitama, 338-8570 Japan; 2 Riken Brain Science Institute, Hirosawa, Wako City, Saitama, 351-0198 Japan; 3 Saitama University Brain Science Institute, 255 Shimo-Okubo, Sakura-ku, Saitama City, Saitama, 338-8570 Japan; 4 Research and Development Bureau, Saitama University, 255 Shimo-Okubo, Sakura-ku, Saitama City, Saitama, 338-8570 Japan; 5 Present Address: Department of Molecular and Cellular Biology, Harvard University, 16 Divinity Avenue, Cambridge, MA 02138 USA

## Abstract

Optical measurement of membrane potentials enables fast, direct and simultaneous detection of membrane potentials from a population of neurons, providing a desirable approach for functional analysis of neuronal circuits. Here, we applied recently developed genetically encoded voltage indicators, ASAP1 (Accelerated Sensor of Action Potentials 1) and QuasAr2 (Quality superior to Arch 2), to zebrafish, an ideal model system for studying neurogenesis. To achieve this, we established transgenic lines which express the voltage sensors, and showed that ASAP1 is expressed in zebrafish neurons. To examine whether neuronal activity could be detected by ASAP1, we performed whole-cerebellum imaging, showing that depolarization was detected widely in the cerebellum and optic tectum upon electrical stimulation. Spontaneous activity in the spinal cord was also detected by ASAP1 imaging at single-cell resolution as well as at the neuronal population level. These responses mostly disappeared following treatment with tetrodotoxin, indicating that ASAP1 enabled optical measurement of neuronal activity in the zebrafish brain. Combining this method with other approaches, such as optogenetics and behavioural analysis may facilitate a deeper understanding of the functional organization of brain circuitry and its development.

## Introduction

During nervous system development, a multitude of neurons are generated and connect with each other to form functional circuits. For a deeper understanding of brain function, it is necessary to uncover the organizing principles as well as the development of such functional circuitry. Recent advances in optical techniques provide powerful tools for analysing functional development, addressing many of the complexities of neuronal circuits. Prominent examples include the optical measurements and control of neuronal activity known as optogenetics^1–4^. These approaches have expanded the functional analysis of neuronal populations, which were previously analysed primarily using electrophysiological recordings^1,5^.

Optical measurement of the membrane potential by voltage sensor imaging is a promising technique enabling fast, direct and simultaneous detection of membrane potentials from a population of neurons^6–8^. Its high speed and directness allow for detection of action potentials and also of hyperpolarization, which are difficult to analyse by imaging with calcium indicators, due in part to the slow kinetics of the indicators and the dynamics of calcium^2,9^. Various types of voltage sensitive dyes (VSDs) have been developed for improved brightness, signal-to-noise ratio, and wider variation in colour^10–12^, and have been used for circuit analysis of the brain, including the cerebellum^13,14^. Among these are red-shifted VSDs that can be combined with optogenetic control of neuronal activity to examine the functional organization of inhibitory circuits^10,15,16^. However, one limitation of VSD imaging is a lack of cell-type specificity, which is critical for the detailed analysis of neuronal circuitry by separating populations of neurons. To overcome this problem, considerable efforts have been made to develop genetically encoded voltage indicators (GEVIs). Recent advances have produced increasing numbers of GEVIs with faster kinetics and brighter fluorescence, overcoming prior weaknesses in both speed and signal to noise ratio^17–21^.

ASAP1 (Accelerated Sensor of Action Potentials 1) is a recently developed GEVI with increased brightness and faster kinetics achieved by using extracellular loops of voltage-sensing domains^20^. Although recent work reported that ASAP1 could not be expressed in zebrafish neurons^22^, limited information is currently available regarding the expression of voltage sensors in zebrafish. QuasAr2 (Quality superior to Arch 2) is a rhodopsin-based and red-shifted sensor with a fluorescence excitation maximum of 590 nm^21^. It is compatible with widely-used optogenetic probes such as channelrhodopsins and with calcium/voltage indicators with excitation peaks at shorter wavelengths, thereby enabling the simultaneous detailed examination of several neuronal populations in the brain. There is currently no report of the successful expression of QuasAr2 in zebrafish brain tissue.

To address how neuronal circuits in the cerebellum are functionally organized and develop, we applied ASAP1 and QuasAr2 to zebrafish. The zebrafish is an appropriate model system for developmental genetics and neuroscience. It is known to have cerebellar circuitry similar to that of mammals^23,24^, and its transparency and small size provide a significant advantage for whole-brain application of optical techniques in circuit analysis^25–28^. Furthermore, transgenic zebrafish lines that express *gal4* in a neuron-type-specific manner are available for most of the major neuron types in the cerebellum, providing an excellent system for circuit analysis^29^. Thus the application of voltage sensor imaging in zebrafish has the potential to provide new insights into the functional development of neuronal circuits, especially in the cerebellum.

In this study, we tested ASAP1 and QuasAr2 in zebrafish brain tissue by generating transgenic zebrafish lines expressing these sensor proteins. By observing their localization in embryos and fluorescence responses to electrical stimulation, we showed that ASAP1 is localized to the cell membrane of neurons, including cerebellar neurons, and detected neuronal activity in the cerebellum by whole-cerebellum imaging. Furthermore, spontaneous activity in spinal cord neurons, as well as evoked responses in the optic tectum, were also detected by ASAP1. In contrast, QuasAr2 could not be detected in zebrafish tissues. This result is an important step forward, and highlights the potential of voltage sensor imaging in the zebrafish model to elucidate the organizing principles of neuronal circuits and their development.

## Results

### Characterization of ASAP1 and QuasAr2 expressions in zebrafish

**Figure 1:**
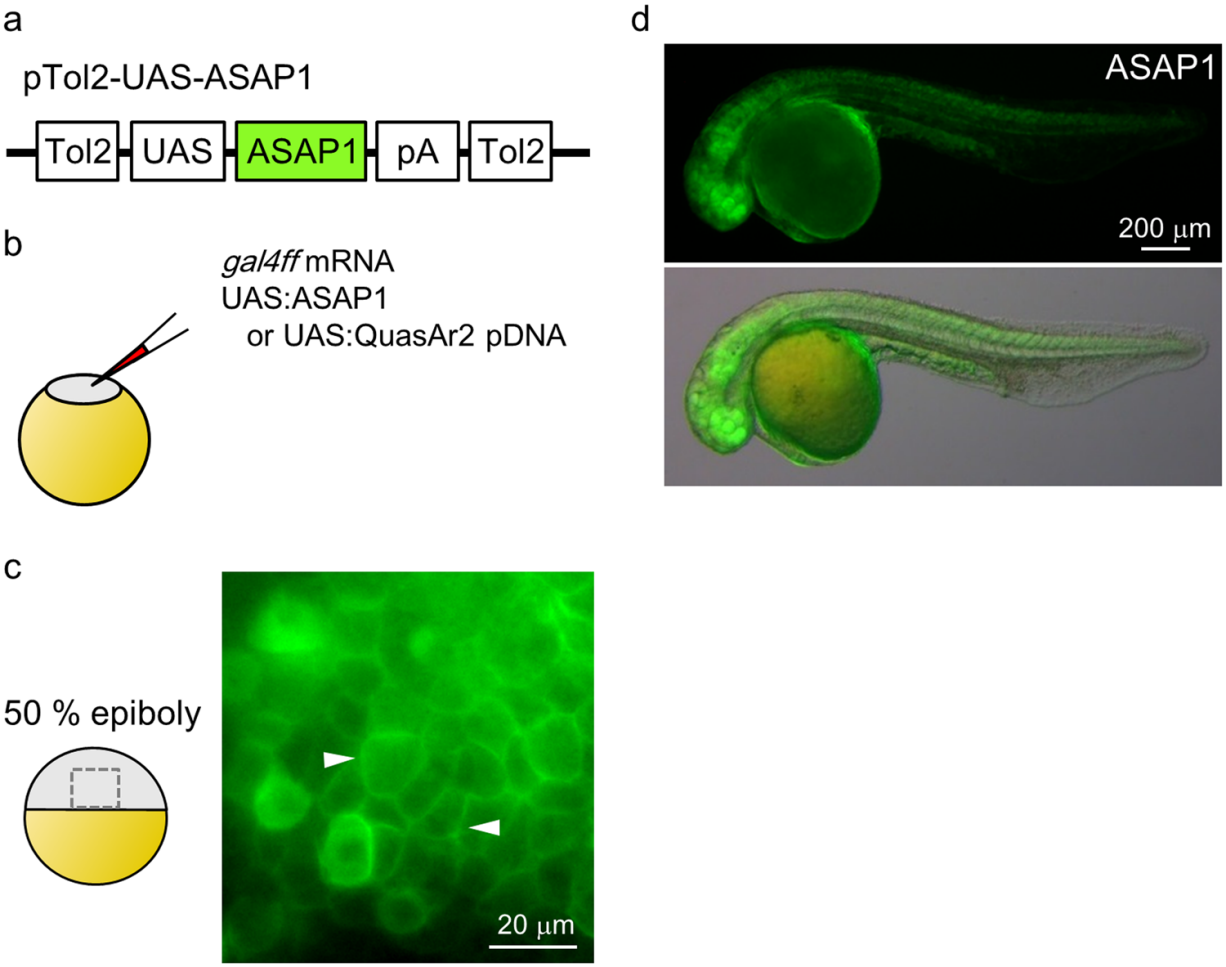
Transient expression of ASAP1. (**a**) Schematic diagram of plasmid construct for ASPA1. (**b**) Schematic diagram of the co-injection experiment. (**c**) ASAP1 was localized to the cellular membranes at 50% epiboly (arrowheads). (**d**) Lateral views of ASAP1-expressing embryos at 1 dpf. ASAP1 was expressed widely in the embryos.

To examine neuronal activity in the zebrafish brain via voltage sensor imaging, two recently developed GEVIs, ASAP1 and QuasAr2, were selected to establish transgenic zebrafish lines using the GAL4-UAS and Tol2 transposon systems^30,31^. First, UAS:ASAP1 and UAS:QuasAr2-mOrange constructs were generated (Fig. 1a, Supplementary Fig. S1a). To test whether these sensors could be expressed in zebrafish, especially in neuronal tissues, the DNA constructs were co-injected with *gal4ff* mRNA into one-cell stage embryos (Fig. 1b). Clear membrane localization of ASAP1 was observed in the injected embryos at the 50% epiboly stage (Fig. 1c). At one dpf (day post-fertilization), ASAP1 was distributed in various regions including the neural tube and optic vesicles (Fig. 1d). In the case of UAS:QuasAr2-mOrange, membrane localization of mOrange fluorescence was also clearly observed in the neuroepithelium (Supplementary Fig. S1b–d). Some neuroprogenitor cells extended mOrange-positive long filaments which may have been filopodia (Supplementary Fig. S1e). However, we failed to detect a near-infrared fluorescence signal attributable to QuasAr2 in any region of the injected embryos (excitation: 638 nm, 3.03 mW/mm^2^, Supplementary Fig. S1b–d).

### *UAS:ASAP1* fish exhibit membrane localization of ASAP1 in the neuroepithelium

**Figure 2:**
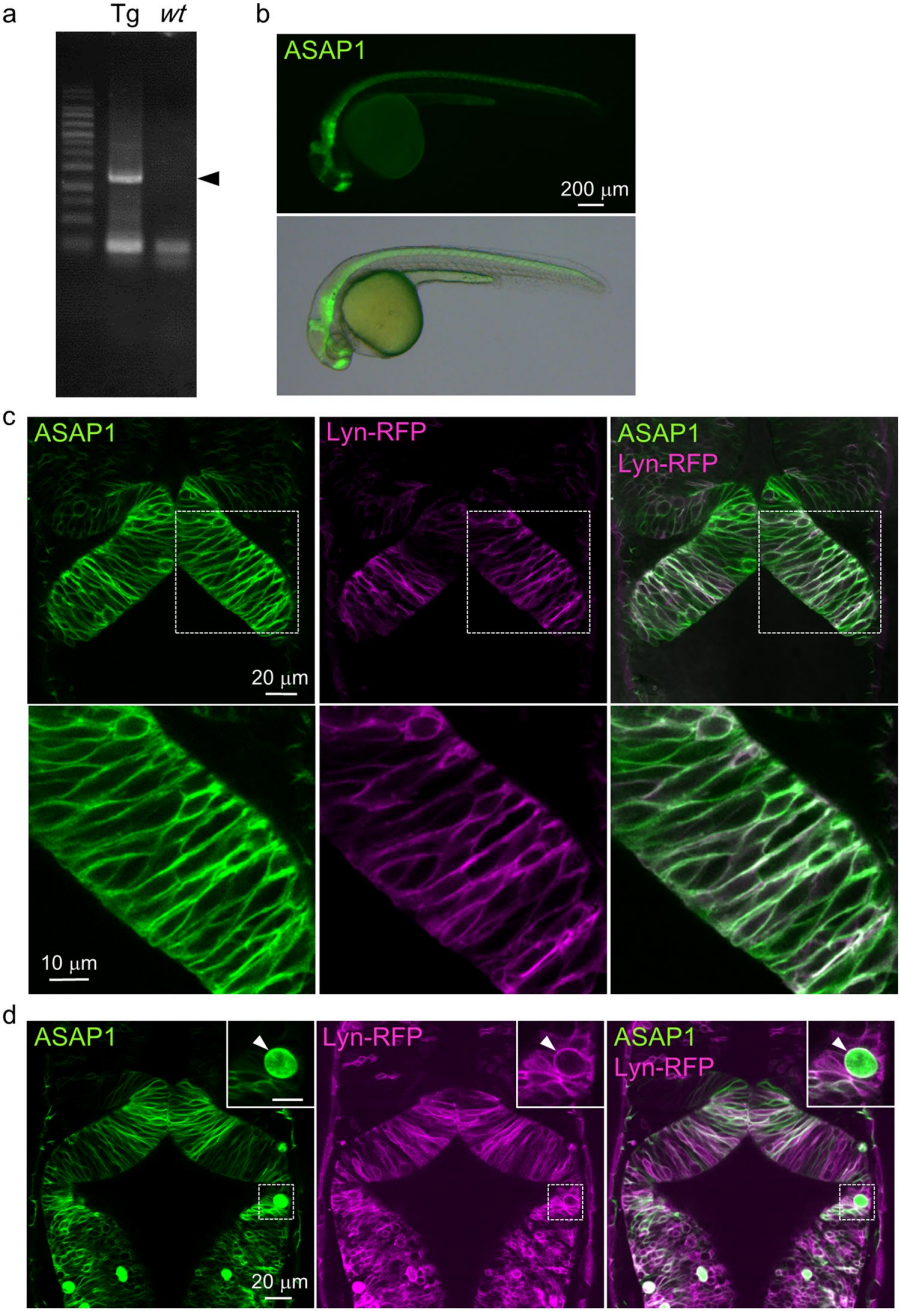
Transgenic zebrafish showed membrane-localized ASAP1 in the neural tube. (**a**) Genotyping results of *Tg(UAS:ASAP1)* fish. Left: *Tg(UAS:ASAP1)* (Tg), right: wild-type *(wt)*. The cropped gel image is shown. The full-length gel is presented in Supplementary Fig. S6a. (**b**) ASAP1 was distributed widely in the neural tube of *Tg(elavl3:GAL4-VP16;UAS:ASAP1)* fish at 1 dpf. (**c**) Dorsal view of the neural tube of *Tg(elavl3:GAL4-VP16;UAS:ASAP1;UAS:lyn-RFP)* embryos at 1 dpf. ASAP1 (green) was co-localized with Lyn-RFP (red: cell membranes) in the neural tube. Higher magnification images are shown in the lower panel. (**d**) Some of the ASAP1 positive cells had irregular shapes (arrowheads). Higher magnification images are shown in the upper right corner.

We next generated transgenic lines expressing the two voltage sensors, and obtained seven and two founder fishes for *UAS:ASAP1* and *UAS:QuasAr2*, respectively (Fig. 2a, Supplementary Fig. S1f).

To examine whether ASAP1 is localized to the cellular membranes of neurons in the *Tg(UAS:ASAP1)* fish, we observed *Tg(elavl3:GAL4-VP16;UAS:ASAP1;UAS:lyn-RFP)* embryos, in which *gal4* was expressed mainly in neurons and the cellular membranes were labelled by Lyn-RFP. At one dpf, ASAP1 was widely observed in the neural tube (Fig. 2b). Confocal imaging of the midbrain region revealed that ASAP1 was co-localized with Lyn-RFP, indicating that ASAP1 was localized to the cellular membrane in the neuroepithelium (Fig. 2c, Supplementary Movie 1). On the other hand, some founder fish showed ASAP1 positive cells with irregularly round shapes (Fig. 2d, n = 6 (6/58, 11.7%)). Based on these observations, two founder fish with higher fluorescence intensity and proper localization of ASAP1 on the membrane were used for the following experiments. In the case of *UAS:QuasAr2-mOrange* fish, however, although several gal4 lines were crossed with *Tg(UAS:QuasAr2-mOrange)* fish, no QuasAr2 or mOrange signal was detected at any stage (n = 107).

### ASAP1 is expressed in neurons, including cerebellar neurons

**Figure 3:**
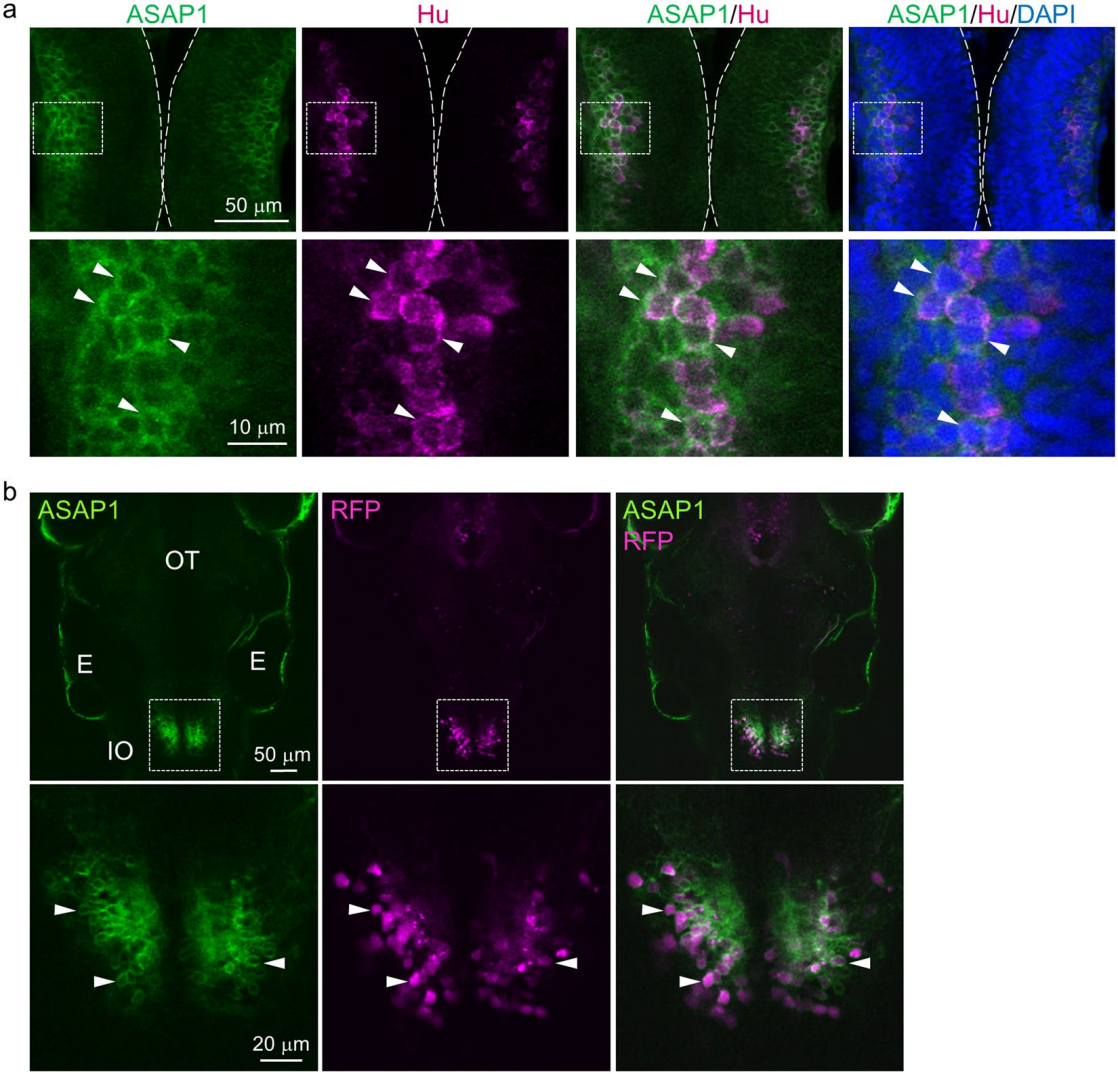
ASAP1 was expressed in neurons, including those of the cerebellum. (**a**) Horizontal sections of the neural tube of *Tg(elavl3:GAL4-VP16;UAS:ASAP1)* embryos stained with a HuC/D antibody and DAPI at 1 dpf. Hu-positive neurons (red) show a membrane-localized ASAP1 signal (arrowheads). Higher magnification images are shown in the lower panel. (**b**) ASAP1 was properly expressed in inferior olivary neurons. Dorsal view of the inferior olive (IO) of *Tg(hspGFFDMC28C;UAS:ASAP1;UAS:RFP)* fish (upper panel). Higher magnification images are shown in the lower panel. ASAP1 (green) is localized to the cell membranes of inferior olivary nuclei (red, arrowheads). Abbreviations indicate optic tectum (OT), and ear (E).

A recent study reported that ASAP1 could not be expressed in zebrafish neurons^22^. In this study, we succeeded in expressing ASAP1 in the neuroepithelium of *Tg(UAS:ASAP1)* fish, and confirmed this immunohistochemically using the pan-neuronal marker Hu (Fig. 3a). At one dpf, all Hu-positive neurons in the hindbrain were found to be positive for ASAP1, indicating that ASAP1 was expressed in the neurons (Fig. 3a, n = 563, 5 fish). Furthermore, ASAP1 expression was examined in cerebellar neurons using several transgenic zebrafish lines which expressed *gal4* specifically in cerebellar neurons^29^. Confocal imaging of zebrafish embryos which express ASAP1 in inferior olivary neurons or cerebellar granule cells revealed that these neurons possessed membrane-localized ASAP1 (Fig. 3b, Supplementary Fig. S2). We also confirmed that ASAP1 was distributed in climbing fibers (Supplementary Fig. S3, Supplementary Movie 2). Thus, we conclude that ASAP1 is specifically expressed in neurons, including cerebellar neurons.

### ASAP1 could detect neuronal activity in zebrafish spinal cord and cerebellum

**Figure 4:**
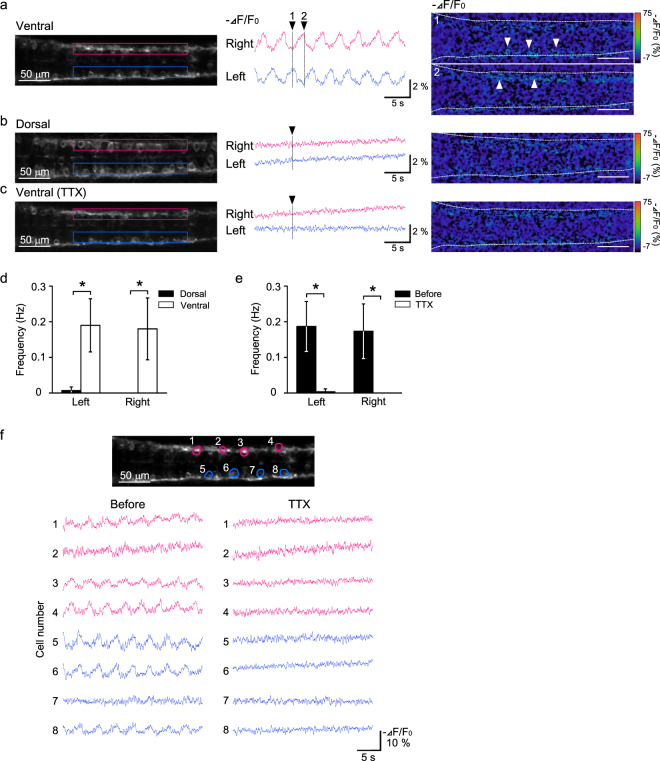
Spontaneous activity of spinal cord neurons was detected via ASAP1 imaging. (**a**) Changes in ASAP1 fluorescence were observed in the ventral region of the spinal cord. (left) Dorsal view of the ventral spinal cord of *Tg (elavl3:GAL4-VP16;UAS:ASAP1)* fish. The rostral side is to the left, and the area between 3–8 somites is shown. Regions of interest (ROIs) located between 5–7 somites are indicated by red (right side) and blue (left side) rectangles. (middle, right) Fluorescence changes of ASAP1 in the ROIs are shown in the middle panel. The images at two time points indicated by black arrowheads are shown in the right panel. White arrowheads indicate the activated neurons. Changes in fluorescence (−ΔF/F0) are indicated by the pseudocolor scale shown at right. (**b**) Fluorescence changes of ASAP1 were not observed in the dorsal region of the spinal cord. (left) Dorsal view of the same embryo shown in (**a**) with the image focus at the ventral region. An image at the time point indicated by the black arrowhead in the middle panel is shown to the right. (**c**) The spontaneous activity in the ventral region of the spinal cord was reduced by tetrodotoxin (TTX) treatment. The imaged plane and ROIs are in the same positions as in (**a**). (**d**) The frequency of the fluorescence changes was significantly higher in the ventral region. Similar results were obtained for both the left and right sides of the spinal cord (6 fish, *p < 0.05). (**e**) The spontaneous activity was almost eliminated by TTX treatment (6 fish, *p < 0.05). (**f**) Monitoring of spontaneous activity of individual cells (cells 1–8) by ASAP1 imaging. (top) A fluorescence image of the ventral spinal cord of *Tg(elavl3:GAL4-VP16;UAS:ASAP1)* fish. ROIs were located at the 8 cells (red: right side, blue: left side). Examples of activity patterns of the 8 cells for two conditions (before and after TTX treatment) are shown in the lower panel.

The applicability of ASAP1 for optical recordings of neuronal activity in zebrafish was examined using *Tg(elavl3:GAL4-VP16;UAS:ASAP1)* fish in which ASAP1 was confirmed to be distributed to the cellular membranes of neurons (Fig. 3). First, we tested whether ASAP1 could detect spontaneous activity of neurons in the spinal cord^32,33^. As shown in Fig. 4, a group of neurons located in the ventral region exhibited oscillatory changes in ASAP1 fluorescence at the frequency of 0.19 ± 0.07 Hz in the left side of the spinal cord and 0.17 ± 0.08 Hz in the right side (6 fish). Phases of the fluorescent changes differed between the left and right sides, and they occurred alternatively (Fig. 4a), which is consistent with previous observations^32,33^. In some cases, fluorescence changes were detected in the vicinity of the cell soma, possibly in the cellular membranes (Fig. 4a, white arrowheads). This activity seldom appeared in the dorsal region (Fig. 4b,d, left: p < 0.05, right: p < 0.05, 6 fish, Mann-Whitney’s U test), and largely disappeared after treatment with the sodium channel blocker tetrodotoxin (TTX) (Fig. 4c,e, left: control 0.19 ± 0.07 Hz, TTX 0.003 ± 0.008 Hz, p < 0.05, right: control 0.17 ± 0.08 Hz, TTX 0 Hz, p < 0.05, Wilcoxon signed-rank test, 6 fish). This tendency was also observed in recordings of individual cells (Fig. 4f). In addition, different patterns of activity were detected for neighbouring cells (Fig. 4f). These results suggest that ASAP1 could detect spontaneous activity of spinal cord neurons at a cellular level as well as at the neuronal population level.

**Figure 5:**
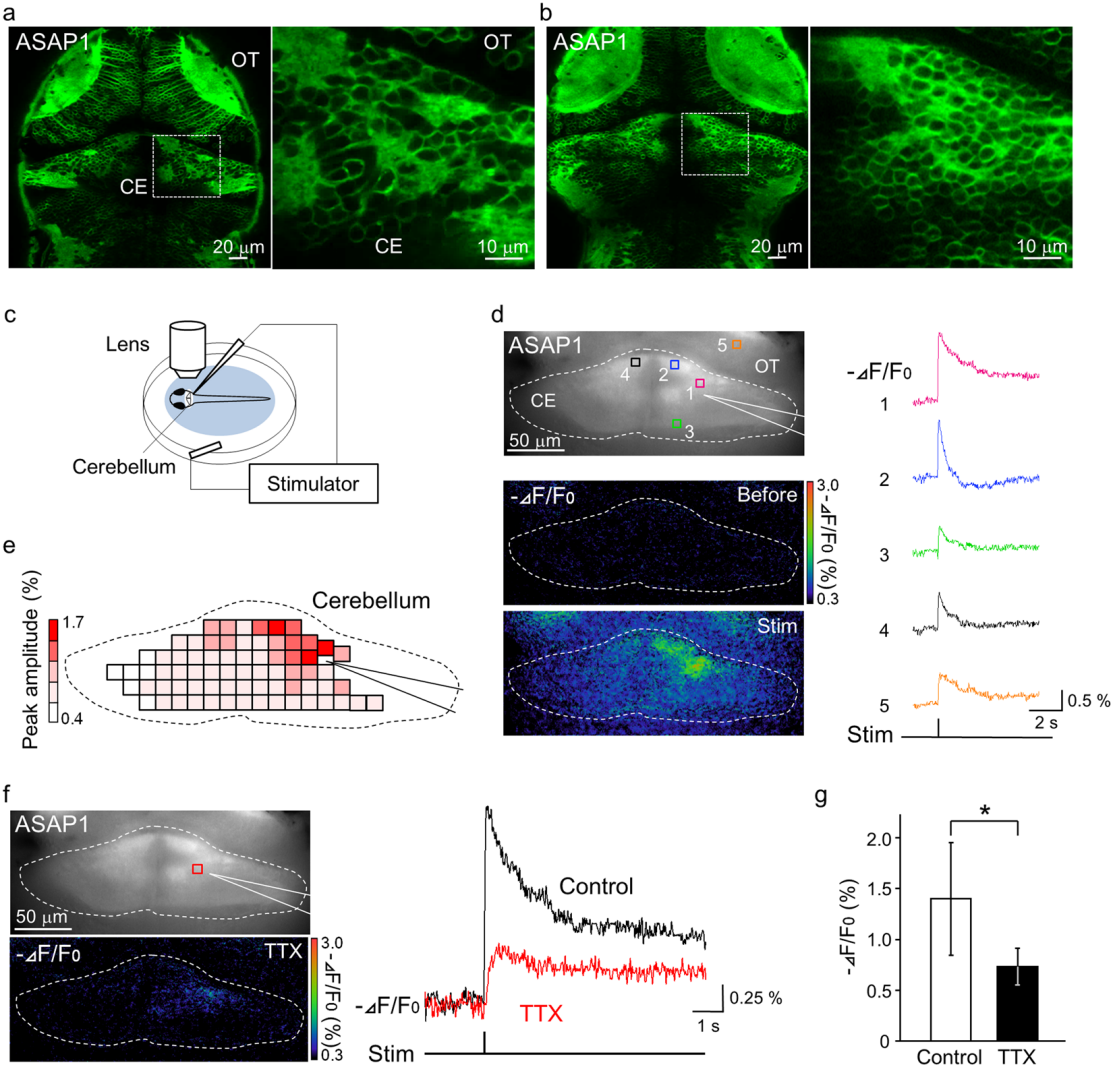
Neuronal activity in the cerebellum and optic tectum was detected by ASAP1 imaging. (**a**,**b**) Membrane-localized ASAP1 was observed in the cerebellum and optic tectum. Dorsal view of *Tg(elavl3:GAL4-VP16;UAS:ASAP1)* fish brain at 6 dpf (**a**) and 9 dpf (**b**). Higher magnification images are shown to the right. CE: cerebellum, OT: optic tectum. (**c**–**e**) The evoked depolarizations in the cerebellum and optic tectum were detected by ASAP1 imaging. (**c**) Schematic diagram of the electrical stimulation system. (**d**) (Top left) A fluorescence image of the cerebellum of *Tg(elavl3:GAL4-VP16;UAS:ASAP1)* fish. (Bottom left) Images of changes in ASAP1 fluorescence before and 0.14 sec after the stimulation. Changes in fluorescence (−ΔF/F0) are indicated by the pseudocolor scale shown at right. (Right) ASAP1 signals produced by electrical stimulation of the cerebellum. Traces indicate signals detected at the 5 numbered locations indicated in the top-left image. ROI5 is located in the optic tectum. (**e**) Response map of the cerebellum. The peak amplitudes of the fluorescence changes in the cerebellar neurons upon stimulation are encoded by the pseudocolor scale shown at left. (**f**) The depolarizing signal measured in the cerebellum was reduced by tetrodotoxin (TTX) treatment (left bottom). Results from the same fish are shown in (**d**) and (**f**). Traces indicate signals detected at the location indicated in the top left image. (**g**) The mean peak values of the responses were significantly reduced after TTX treatment (*p < 0.01). This indicates that the depolarizing response represents neuronal activity in the cerebellum and optic tectum.

Next, ASAP1 imaging was performed in the cerebellum using *Tg(elavl3:GAL4-VP16;UAS:ASAP1)* fish, which had ASAP1 localized to the membrane (Fig. 5a,b, Supplementary Movie 3). After five dpf, when the basic structure of the cerebellar circuits was formed^23^, the cerebellum was electrically stimulated using a glass pipette located on the cerebellum, and the resulting neuronal activity was detected by high-speed imaging (Fig. 5c). Upon stimulation, changes in ASAP1 fluorescence were widely observed in the cerebellum (mean peak value: 1.23%, standard deviation (s.d.): 0.49, n = 15, 45 trials, Fig. 5d,e, Supplementary Movie 4). Depolarizing responses were prominent in the vicinity of the stimulated position, in a rostral region of the contralateral hemisphere, and in a caudal region of the cerebellum. This caudal region of the cerebellum is assumed to be an area which contains many axons (Supplementary Fig. S4). Depolarization was also detected in the optic tectum (Fig. 5d). This evoked activity was significantly reduced following treatment with TTX by 41.6 ± 21.3% (Fig. 5f,g,p = 0.0097 by paired t-test, n = 7, 21 trials for the control and TTX conditions, respectively; mean peak value: control 1.4%, TTX 0.73%, s.d: control 0.55, TTX 0.17). These results indicate that the depolarizing signals were neuronal responses. Simultaneous recordings of membrane potential by whole-cell patch-clamp recordings and ASAP1 imaging showed that the recorded neurons were indeed depolarized (5 cells, 5 fish, Supplementary Fig. S5a). Further analysis of the sensitivity and temporal resolution of the evoked responses showed a roughly proportional increase of the response size relative to the stimulus intensity (Supplementary Fig. S5b), and also showed the response speed (time to peak: 180.4 ± 111.4 ms, onset time: 7.97 ± 7.13 ms, 15 fish, 45 trials). The rather slow responses could be a consequence of the fact that we observed responses of a population of neurons in the cerebellum, where most of the neurons expressed ASAP1 as shown in Fig. 5. We also compared the response patterns to trains of stimulation at different frequency, showing that for frequencies above 5 Hz, it was difficult to detect a response following each stimulation pulse (Supplementary Fig. S5c).

Taking these results together, it is concluded that ASAP1 enables voltage sensor imaging of neuronal activity in zebrafish brain circuitry.

## Discussion

Optical measurement of membrane potentials is a desirable approach for functional analysis of neuronal circuits that contain both excitatory and inhibitory neurons. Recent advances in the development of GEVIs have overcome some of their initial weaknesses, including low signal to noise ratio and limited speed, by obtaining higher fluorescence intensity and faster kinetics. In the present work, we took advantage of zebrafish for optical techniques and sought to employ two newly developed voltage sensors, ASAP1 and QuasAr2. By establishing transgenic zebrafish lines and characterizing these sensors in the zebrafish brain *in vivo*, we showed that, in contrast to previous reports, ASAP1 could indeed be expressed in zebrafish neurons, including cerebellar neurons, and also that neuronal activity in the zebrafish spinal cord, cerebellum, and optic tectum could be detected by ASAP1. Our current work provides an important step forward for applying voltage sensor imaging in zebrafish to elucidate the organizing principles of neuronal circuitries.

In the present study, we successfully established transgenic zebrafish lines expressing ASAP1 and QuasAr2, and used them to characterize these recently developed GEVIs. We could clearly observe membrane localized ASAP1 signals in almost all neurons examined, including cerebellar neurons, which is in contrast to the previous investigation^22^. Possible causes might be a difference in the promoters that were used or positional effects related to the transgene integration site. Some ASAP1 transgenic fish had ASAP1-positive neurons with defective shapes and a stronger ASAP1 signal, even detected in the cytoplasm (two founder fish). The defective cell shapes might be a result of this excessive expression.

QuasAr2 has a red-shifted excitation peak which makes it a promising choice for circuit analysis, by allowing deeper penetration of light into the neuronal tissue. It can also be combined with other GEVIs/calcium indicators or optogenetic actuators that have excitation peaks at shorter wavelengths. We were able to establish *Tg(UAS:QuasAr2-mOrange)* zebrafish, but we could not detect any QuasAr2 signal at any stage. In embryos which were co-injected with *gal4ff* mRNA and UAS:QuasAr2-mOrange pDNA, we only observed mOrange fluorescence signals in the cellular membranes, showing that the mOrange protein was properly targeted to the membrane. Further efforts, such as increasing the expression level of QuasAr2 or the application of electrochromic FRET imaging,^34^ which utilizes voltage-dependent nonradiative quenching of mOrange, could help to overcome this problem.

To test whether ASAP1 enables detection of neuronal activity in zebrafish brain, we measured optical signals in the cerebellum and optic tectum in response to direct electrical stimulation of the cerebellum. Three lines of evidences show that neuronal activity was detected by ASAP1 imaging. First, clear depolarizing responses were observed widely in the cerebellum and optic tectum upon stimulation. Second, the spatial pattern of these responses matched the neuronal structure of the cerebellum. Third, the evoked responses were reduced by treatment with tetrodotoxin, a sodium channel blocker. Furthermore, we succeeded in recording the spontaneous activity of spinal cord neurons at single-cell resolution as well as at the neuronal population level. We also noticed that some spinal cord neurons showed different patterns of activity from others, which might be a consequence of the heterogeneous properties of these neurons. To our knowledge, this is the first report showing that ASAP1 could detect neuronal activity in zebrafish brain and be used for the functional analysis of the neuronal circuits.

There are, however, some limitations. For circuit analysis of the brain, measuring neuronal activity at single cell resolution is optimal. With the ASAP1 signals observed here, we succeeded in detecting cellular responses in the spinal cord, but it was difficult to isolate single-cell responses in the cerebellum. This may partly be due to the fact that voltage indicators detect the sum of excitatory and inhibitory signals, thereby possibly reducing the size of the depolarizing signals observed in this experiment, which could be more prominent in the cerebellum with a number of inhibitory inputs. Another reason may line in the properties of ASAP1 whose fluorescence intensity is reduced upon depolarization. In the specimens without sufficient ASAP1 expression, this could reduce the signal to nose ratio (SNR). Indeed, the fluorescence intensity of ASAP1 tended to be higher in the spinal cord compared to the cerebellum. Thus, the SNR might be improved by using transgenic fish which have higher expression of ASAP1, but distributed more sparsely, or by using ASAP3 which shows improved response size and sensitivity.

In conclusion, we have characterized two newly developed GEVIs, ASAP1 and QuasAr2, in zebrafish brain tissue by establishing transgenic fish lines, and showed that ASAP1 is a promising indicator for analysis of functional circuits in zebrafish, a highly advantageous model animal for optical approaches and developmental biology. Further improvements in this kind of functional imaging could help to reveal functional properties of brain circuitries, especially focusing on the timing of neuronal activity and inhibitory circuits which are known to play important roles in the cerebellum. This could also be used to analyse the membrane potentials of various cell types, including non-neural cells, which are involved in development. Furthermore, combining ASAP1 imaging with other techniques, such as optogenetic control or detection of neuronal activity via red-shifted optogenetic probes^35,36^, and behavioural tests, could deepen our understanding of the functional organization of the brain and its development.

## Methods

### Fish lines

Wild-type zebrafish (*Danio rerio*) with the RW genetic background were mainly used. To generate pTol2-UAS-ASAP1 and pTol2-UAS-QuaesAr2-mOrange2 plasmid, *ASAP1* and *QuasAr2-mOrange2* sequences were excited from pcDNA3.1/Puro-CAG-ASAP1 and AAV-CaMKlla-QuasAr2-mO2 plasmid (Addgene), respectively, and inserted into pTol2-UAS-MCSF-polyA, in which a UAS and polyA sequence had been integrated into the pTol2-MCS plasmid^37^. To make Tg lines, Tol2 plasmid DNA and *transposase* mRNA were co-injected into one-cell stage embryos. Genotyping was performed using the following primers: *ASAP1* (5′-TCCAAACTGAATACTTTGGATG-3′, 5′-CACCTCCCCCTGAACCTGAAACATA-3′), *QuasAr2* (5′-ATCTACCTTTAACACCCTGACA-3′, 5′-CACCTCCCCCTGAACCTGAAACATA-3′). *Tg(UAS;lyn-RFP)* line was generated using Tol2-UAS:lyn-RFP plasmid DNA^38^. *Tg(elavl3:GAL4-VP16)*, *Tg(hspGFFDMC28C;UAS:RFP)* and *Tg(hspGFFDMC152B;UAS:RFP)* fish lines were described previously^29,39^. For live imaging and immunohistochemistry studies, zebrafish embryos were treated with 0.005% phenylthiourea (PTU). Some Tg lines were crossed with nacre^40^. All procedures were performed in accordance with a protocol approved by the Saitama University Committee on Animal Research.

### Injection of plasmid DNA and mRNA

20 or 25 ng/μl of plasmid DNA and 20 ng/μl of *gal4ff* mRNA were co-injected into one-cell stage embryos. ASAP1 or QuasAr2 expression was examined at 50% epiboly and 1 dpf stage.

### Confocal imaging

Zebrafish embryos were paralyzed in 0.02% tricaine, and mounted on 2% methylcellulose or 1% low melting-point agarose. Optical sectioning was performed with Olympus FV1000 or NIKON A1R (detector: A1-DU4) confocal microscopes. For image analysis, ImageJ, NIS-Elements (Nikon), and Imaris (Bitplane) software were used.

### Immunostaining

Whole-mount immunostaining of zebrafish embryos was performed as described previously^41^. Anti-Hu C/D antibody (BD, 1:500) was used as a primary antibody, and Alexa Fluor 555 goat anti-mouse IgG antibody (Molecular Probes) was used as a secondary antibody. Nuclei were counterstained with DAPI (Molecular Probes). These specimens were observed by a confocal microscope (Olympus FV1000).

### Voltage sensor imaging of the spinal cord

Embryos from 20 to 23 hpf were paralyzed with tubocurarine (0.5 mM, Sigma), with their tail tips cut for better penetration of tubocurarine as described previously^32,33^. The embryos were then mounted on 1.8% low melting-point agarose (Sigma) and 0.1 mM tubocurarine. The agarose around their tail was removed to allow efficient transfer of the drug solution (1 µM TTX, TOCRIS, 30 mins). High-speed confocal scanning was performed using a confocal microscope (NIKON A1R) with a 40×/0.8 NA water-immersion lens. Fluorescence images were obtained at around 120 Hz and analysed using NIS-Elements (NIKON) and ImageJ programs. The peaks whose amplitude is higher than 1% were detected manually by using NIS-Elements, and analysed using statcel3 program (Bell Curve).

### Voltage sensor imaging of the cerebellum

Embryos from 5 to 9 dpf were paralyzed with tricaine (0.02%, Sigma) and tubocurarine (0.1 mM, Sigma), and transferred to an extracellular solution (in mM: 134 NaCl, 2.9 KCl, 2.1 CaCl_2_, 1.2 MgCl_2_, 10 HEPES, and 10 glucose, adjusted to pH 7.8 with NaOH) that contained 0.01 mM tubocurarine. Embryos were then mounted on 2% low melting-point agarose (Sigma). The skin above the cerebellum was carefully removed using fine forceps to expose the brain. For imaging, a fluorescence microscope (FN-1, NIKON) equipped with a CMOS camera (ORCA-Flash4.0; Hamamatsu photonics) was used with a 40×/0.8 NA water-immersion lens. Fluorescence images were acquired with HCImage or HSR software (Hamamatsu photonics) at around 100 Hz. To improve the signal-to-noise ratio, images from three trials were averaged. Images were analysed with either NIS-Elements (NIKON) or Fiji programs. For drug treatment, tetrodotoxin (1 μM) was added to the extracellular solution. All experiments were performed at room temperature.

### Electrical stimulation and electrophysiological recordings

Optical responses were evoked by electrical stimulation via a glass pipette filled with the external solution. Current pulses (20 pulses of 3 mA current with 1 ms duration at 33 Hz in most of the cases) were generated by an Electronic Stimulator (Nihon Kohden). For some experiments, protocols with different intensities and frequencies were used. Whole-cell patch clamp recordings were made as described previously at room temperature^42,43^. An internal solution consisting of (in mM): 110 K-gluconate, 15 KCl, 2 MgCl_2_, 4 Na2-ATP, 10 HEPES, and 10 EGTA (pH 7.2) was used. Electrical responses were acquired via a CEZ-2400 amplifier (Nihon Kohden) and DAQ interface (National Instruments, USB-6212 BNC), with WinWCP software (Univ. of Strathclyde).

## Electronic supplementary material

Supplementary Figures

Supplementary Movie 1

Supplementary Movie 2

Supplementary Movie 3

Supplementary Movie 4
