# Supplementary material for: Optical interrogation of neuronal circuitry in zebrafish using genetically encoded voltage indicators
Source: Sci Rep. 2018 Apr 16;8:6048. doi: 10.1038/s41598-018-23906-1 (PMC5902623; doi:10.1038/s41598-018-23906-1)
Supplement: Supplementary file 1 — Supplementary Figures [file 41598_2018_23906_MOESM1_ESM.pdf]

Optical interrogation of neuronal circuitry in zebrafish using genetically encoded voltage indicators

Hiroaki Miyazawa[1]*, Kanoko Okumura[1]* Kanae Hiroshi[1]*, Kazuhiro Maruyama[1], Hisaya Kakinuma[2], Ryunosuke Amo[2#], Hitoshi Okamoto[2], Kyo Yamasu[1,3], Sachiko Tsuda[1,3,4 §]

1 Division of Life Science, Graduate School of Science and Engineering, Saitama University, 255 Shimo-Okubo, Sakura-ku, Saitama City, Saitama 338-8570, Japan.
2 Riken Brain Science Institute, Hirosawa, Wako City, Saitama 351-0198, Japan
3 Saitama University Brain Science Institute, 255 Shimo-Okubo, Sakura-ku, Saitama City, Saitama 338-8570, Japan.
4 Research and Development Bureau, Saitama University, 255 Shimo-Okubo, Sakura-ku, Saitama City, Saitama 338-8570, Japan.

# Present address: Department of Molecular and Cellular Biology, Harvard University, 16 Divinity Avenue, Cambridge, MA 02138, USA.
* These authors contributed equally to this work.
§ Author for correspondence (stsuda@mail.saitama-u.ac.jp)

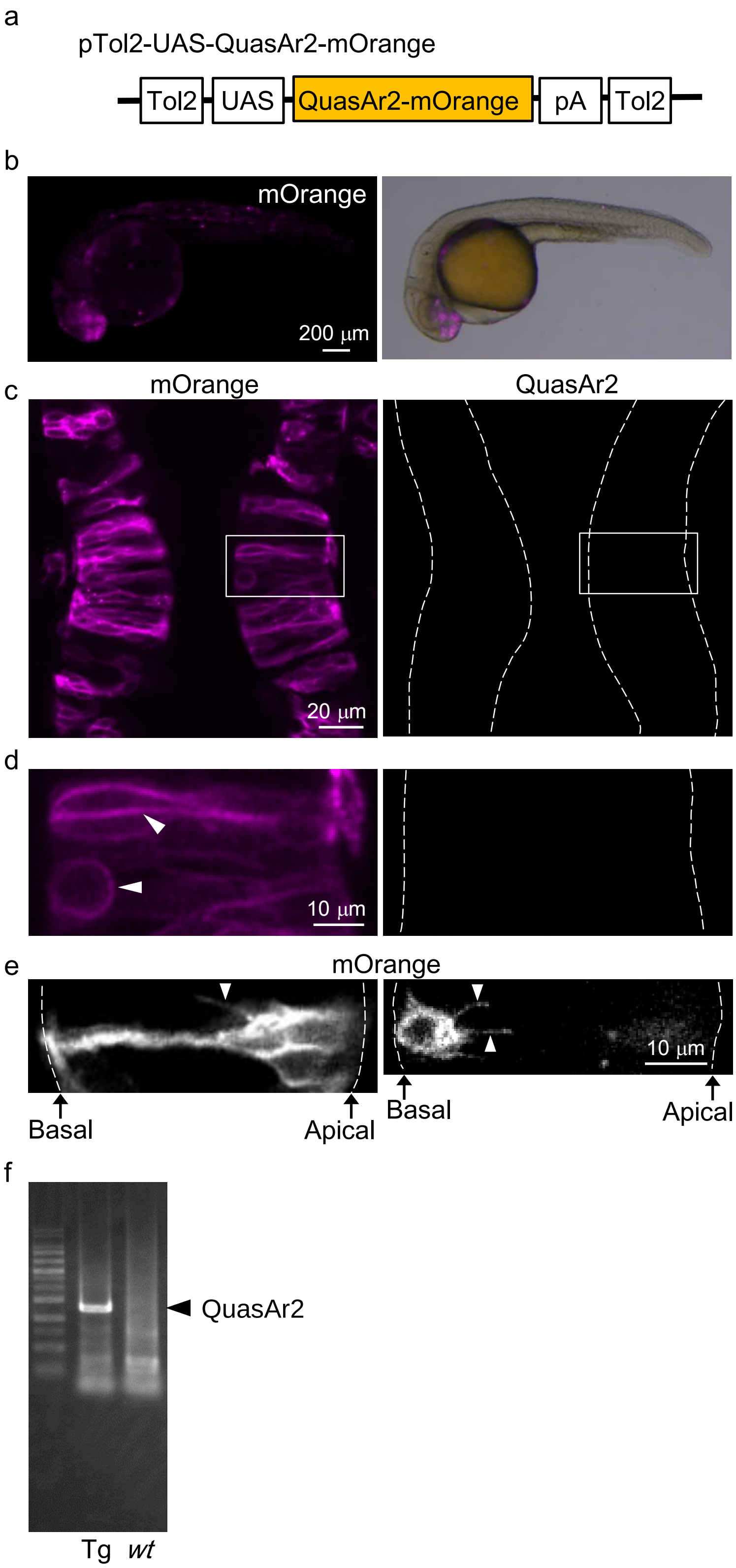
a
pTol2-UAS-QuasAr2-mOrange
Tol2
UAS
QuasAr2-mOrange
pA
Tol2
b
mOrange
200 μm
c
mOrange
QuasAr2
20 μm
d
10 μm
e
mOrange
10 μm
Basal
Apical
Basal
Apical
f
QuasAr2
Tg
wt

Supplementary Figure S1

Transient expression of QuasAr2 and generation of *QuasAr2* transgenic line. (a) Schematic diagrams of plasmid constructs for *QuasAr2*. (b) Lateral views of mOrange-expressing embryos at 1 dpf. (c) mOrange2 was localized to cell membranes in the neural tube, but QuasAr2 was not detected. Dorsal view of the neural tube of the injected embryos. (d) High magnification images of (c). Arrowheads indicate the localization of the fluorescence signal in cell membrane. (e) Representative images of the filopodia which are mOrange positive (arrowheads). (f) Genotyping results of *UAS:QuasAr2* transgenic zebrafish. Tg: *Tg(UAS:QuasAr2)*, *wt*: *wild-type*. gDNA isolated from the embryos of *Tg(UAS:QuasAr2)* F0 fish crossed with *wild-type* fish was used as the template for Tg. The cropped gel image is shown. The full-length gel is presented in Supplementary Fig. S6.

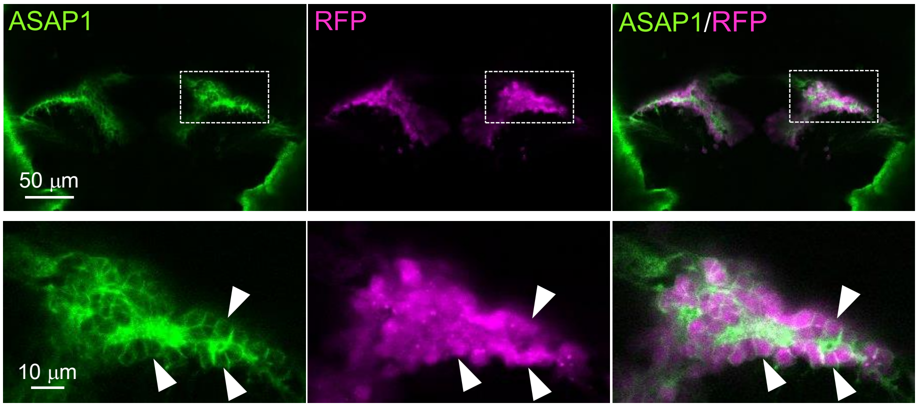

Supplementary Figure S2

ASAP1 (green) is distributed in cerebellar granule cells (red). Dorsal view of the cerebellum of *Tg(hspGFFDMC152B;UAS:RFP;UAS:ASAP1)* fish observed at 5 dpf. Higher magnification views are shown in the lower panel.

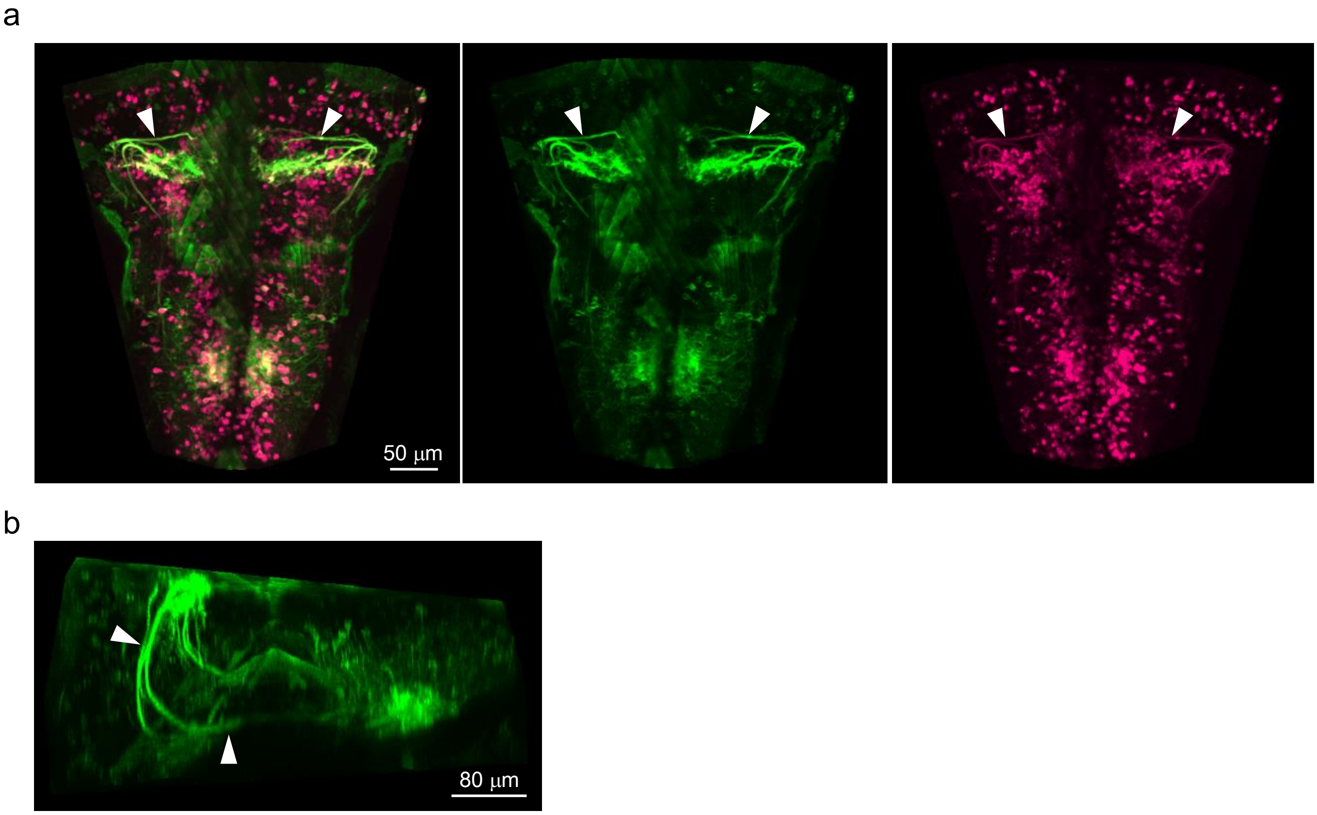

Supplementary Figure S3

3D images of *Tg(hspGFFDMC28C;UAS:ASAP1)* fish observed at 6 dpf. ASAP1 (green) expression was observed in climbing fibers (arrowheads). a: dorsal view, b: left view.

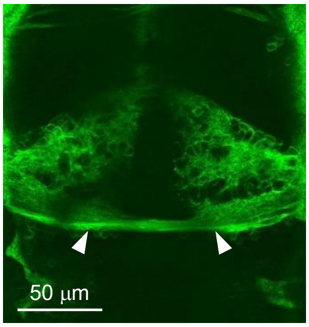

Supplementary Figure S4

A confocal image (z-stack) of the cerebellar region of *Tg(elavl3:GAL4-VP16; UAS:ASAP1)* fish at 5 dpf. Dorsal view is shown. Axon bundles (arrowheads) were observed at the caudal region of the cerebellum.

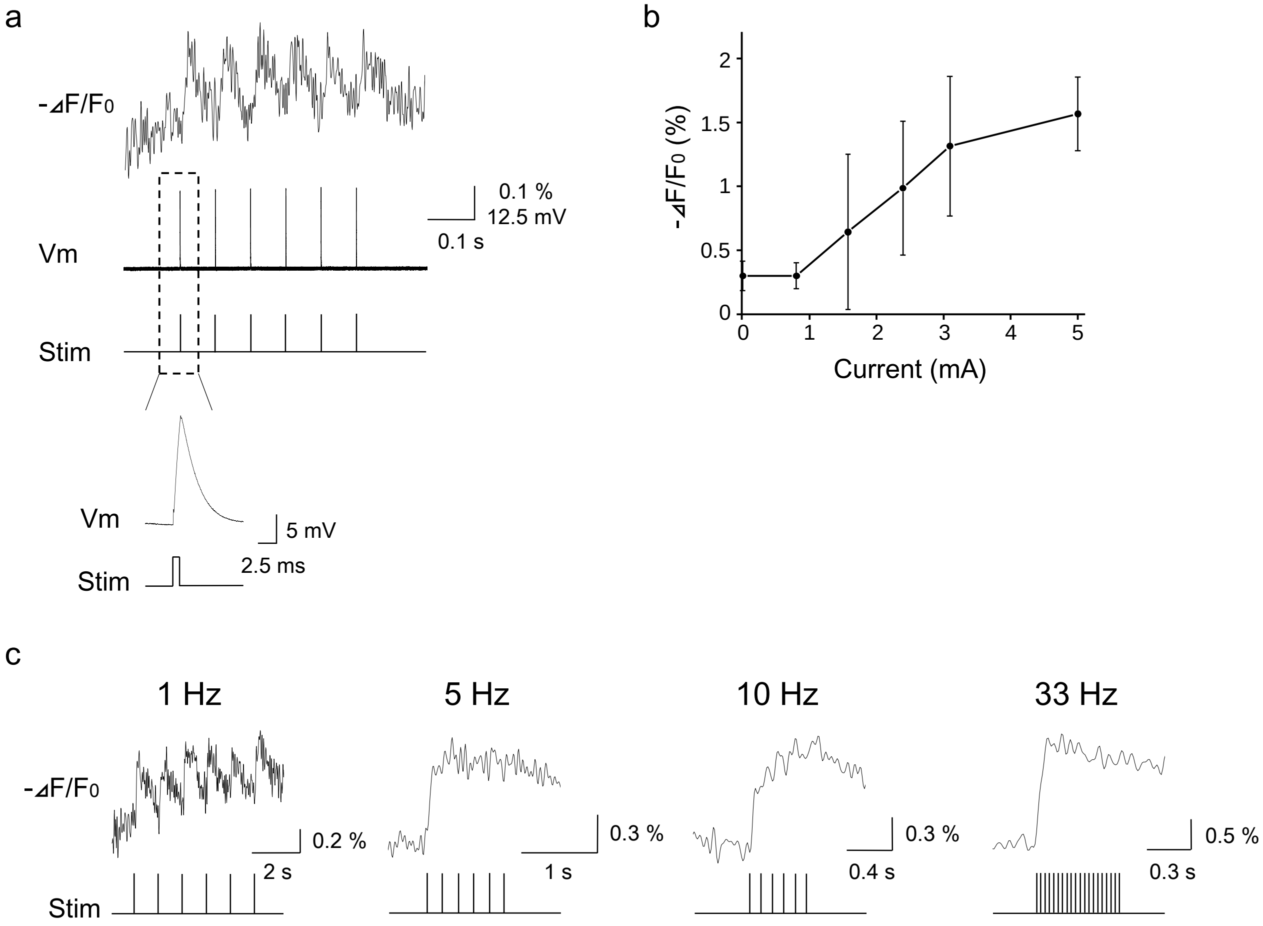

Supplementary Figure S5

Monitoring depolarization in the cerebellum with electrical stimulation at different frequency and intensity. (a) Simultaneous recording of the optical signal and the membrane potential showed evoked responses upon electrical stimulation (3.1 mA, 1 ms, 1 Hz). A higher magnification image is shown in the lower panel. (b) The peak amplitude of the fluorescence changes increased along with the stimulation intensity (0 mA to 3.1 mA: 7 fish, 7 trials, 5 mA: 3 fish, 3 trials). (c) Representative traces of ASAP1 fluorescence change ($-\Delta F/F_0$) at different frequency stimulation.

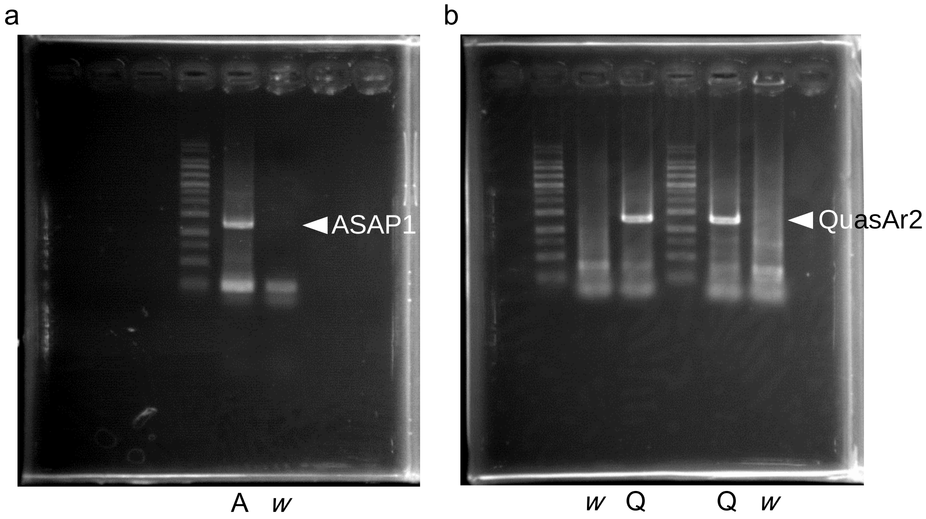

Supplementary Figure S6

The entire gel images of the cropped data shown in Fig. 2a (a) and Supplementary Fig. S1f (b). Genotyping results of (a) *Tg(UAS:ASAP1)* and (b) *Tg(UAS:QuasAr2)* transgenic zebrafish. A: *Tg(UAS:ASAP1)*, Q: *Tg(UAS:QuasAr2)*, *w*: *wild-type*.

Supplementary Movie 1

An optical sectioning of the hindbrain region of *Tg(elavl3:GAL4-VP16;UAS:ASAP1)* fish at 1 dpf. ASAP1 is localized to the cell membranes of the zebrafish neurons.

Supplementary Movie 2

3D reconstruction of the cerebellar region of *Tg(hspGFFDMC28U;UAS:ASAP1)* fish at 6 dpf. ASAP1 (green) is localized to the climbing fibers (red).

Supplementary Movie 3

An optical sectioning of the cerebellum of *Tg(elavl3:GAL4-VP16;UAS:ASAP1)* fish at 6 dpf. ASAP1 is localized to the cell membranes of the zebrafish neurons.

Supplementary Movie 4

Depolarization was observed widely in the cerebellum and optic tectum of *Tg(elavl3:GAL4-VP16;UAS:ASAP1)* fish at 6 dpf upon electrical stimulation. Simulation was performed at 0.96 sec. The movie was acquired at a 100 Hz frame rate.
